# Antifungal Effect of Lavender Essential Oil (*Lavandula angustifolia*) and Clotrimazole on *Candida albicans*: An *In Vitro* Study

**DOI:** 10.1155/2015/261397

**Published:** 2015-10-13

**Authors:** Fereshteh Behmanesh, Hajar Pasha, Ali Asghar Sefidgar, Mohsen Taghizadeh, Ali Akbar Moghadamnia, Hajar Adib Rad, Leyla Shirkhani

**Affiliations:** ^1^Department of Midwifery, Faculty of Medicine, Babol University of Medical Sciences, Babol, Iran; ^2^Infertility and Reproductive Health Research Center, Health Research Institute, Babol University of Medical Sciences, Babol, Iran; ^3^Department of Parasitology and Microbiology, Babol University of Medical Sciences, Babol, Iran; ^4^Research Center for Biochemistry and Nutrition in Metabolic Diseases, Kashan University of Medical Sciences, Kashan, Iran; ^5^Neuroscience Research Center, Department of Pharmacology, Babol University of Medical Sciences, Babol, Iran; ^6^Department of Mycology and Parasitology, Faculty of Medicine, Babol University of Medical Sciences, Babol, Iran

## Abstract

*Background*. The treatment of candidiasis infections is an important problem in the health care system. This study aimed to investigate the* in vitro* effect of lavender essential oil and clotrimazole on isolated* C. albicans* from vaginal candidiasis.* Materials and Methods*. In this clinical trial,* C. albicans* isolated from the vaginal discharge samples was obtained.* Results*. The pairwise comparison showed that lavender and clotrimazole had a significant difference; this difference in the lavender group was lower than clotrimazole. But, after 48 hours, there was no difference seen between groups. There was a significant difference between clotrimazole and DMSO groups. Comparing the changes between groups based on the same dilution, at 24 h and 48 h in clotrimazole group, showed a significant difference two times in the fungal cell count that its average during 48 h was less than 24 h. A significant difference was observed between the two periods in lavender group, only at the dilutions of 1/20 and 1/80. The average fungal cell count after 48 h was also lower in lavender group.* Conclusions*. Given that the lavender has antifungal activity, this can be used as an antifungal agent. However, more clinical studies are necessary to validate its use in candida infection.

## 1. Introduction

Gynecologic infections are the most common reasons women visit a doctor and ask to be treated [1] and, in recent years, the severity and incidence of fungal infections have increased surprisingly. One of the diseases caused by vaginal endogenous flora is candidiasis. It is estimated that 75% of women experience vulvar-vaginal candidiasis at least once in their lifetime. Almost 45% of women are suffering from this infection twice every year. Fortunately, only a small number of women are suffering from chronic recurrent infections.* C. albicans* is the responsible germ in 85–90% of vaginal yeast infections [2]. Early diagnosis and appropriate treatment of vaginitis are important, because failure to timely and appropriately treat these infections leads to serious complications such as pelvic inflammatory disease, infertility, chronic pelvic pain, premature birth, and the dangers of HIV infection [3]. Since the number of patients with AIDS and diabetes and the use of antibiotics increase every day and given that this fungal disease has a high prevalence in this patient population, it is important to find a safe and also a proper treatment. Oily essence has traditionally been used in the treatment and the prevention of various diseases and infections [4].

In the present era, a big trend in medical care in America has occurred which is the use of complementary and alternative medicine. It includes a range of treatments from acupuncture and prayer meetings and, more importantly, the use of herbal medicines which include the traditional herbs and native plants from China, India, Africa, Europe, and America. Although basic scientific research has not been done on these plants, the background of using herbs during the past centuries, as well as being a natural product, has proven that it is a relatively safe therapy. So, this alternative medicine has been emphasized because of the limited side effects and low cost for its application in chronic diseases therapy and prevention [5].

Lavender (*Lavandula angustifolia*), as a kind of medicinal herb, can be effectively used in the treatment of vaginal discharges. Lavender essential oil is frequently used in traditional medicine [6] and its impact on reducing the amount of* Candida albicans* fungus has been shown in* in vitro* [7] and clinical studies [8].

On the other hand, one of the conventional chemical drugs to treat vaginal candidiasis is clotrimazole. Since this chemical drug has side effects, such as increased liver enzymes, painful urination, and depression (due to systemic absorption of the drug), and complications such as irritation and feeling of tingling or contact dermatitis [9], and due to the increasing resistance to antifungal drugs [10], in this study, the effect of lavender (*Lavandula angustifolia*) and clotrimazole on the vaginal candidiasis growth,* in vitro*, was assessed.

## 2. Methodology

In this study, sampling was done vaginally in sterile conditions from 45 women with clinical symptoms of vaginal fungal infection who referred to clinics affiliated to Babol University of Medical Sciences. After placing the patient in a lithotomy position and putting a sterile speculum into the vagina, a sterile cotton swab was used for sampling from the posterior fornix and the lateral sides of the vagina, and the sample was then placed in a sterile tube. The samples were tested and delivered to the Mycology Laboratory of Babol University of Medical Sciences. Only 20 out of the 45 samples were positive in* in vitro* test. The referenced methods used were the cultivation environment of Candida CHROMagar, formed germ tube in human serum, and corn meal agar for making chlamydospore in this environment.

In the laboratory, wet smear was done to detect the yeast fungal cells and trichomonas infections; besides, laboratory culture on Sabouraud, BHI medium for primary isolation of fungi and germ tube test by human blood serum with CHROMagar to identify the types of candida were conducted. The fungi were maintained and passaged in Sabouraud medium for the* in vitro* evaluation of clotrimazole 1% and the oily essence of* lavender* on the* C. albicans* was examined in over 20 samples isolated from 45 patients, using cell count (macrodilution) method. Clotrimazole drop is one of the main drugs that is used to treat fungal infections in Iran and Behvazan Company is one of several pharmaceutical companies in Iran that produces and distributes this drug. Clotrimazole drop was purchased from a pharmacy in Babol, while the lavender essential oil was prepared and purchased from Barij Essence Company. In this company, the aerial parts of lavender plant were used and its essential oil was taken by* Clevenger*. The compounds of* Lavandula angustifolia* after GC (Gas Chromatography) were limonene, cineole, linalool, and linalyl acet. It is not clear which one of them has antifungal effect. It seems that the total compounds have antifungal effect.

In macrodilution method, a loopful colony pure culture of* C. albicans* on Sabouraud dextrose agar provided a 0.5 McF* suspension* of candidal concentration (10³ and 10^6^ fungal cells per mL) in BHI (Brain Heart Infusion) agar broth culture medium.

After preparing the half-McFarland suspension of the* C. albicans*, lavender and clotrimazole dilution was performed. Thus, lavender with pure solvent DMSO (*Dimethyl Sulfoxide*) was diluted to 1/5 in a sterile tube, and the same procedure was also performed for clotrimazole. Then, the dilutions of 1/10, 1/20, 1/40, 1/80, and 1/160 of each drug were prepared and poured in 5 wells of a microplate. In the sixth well of a microplate, the DMSO control was poured and, ultimately, 100 microliters of half-McFarland suspension of fungi was added to all the wells of a microplate. This procedure was performed three times for lavender and three times for clotrimazole (in triplicate) and, finally, the microplates were placed in a bain marie at 37°C. After 24 and 48 hrs, the fungal cells were counted using a microscope Neubauer hemocytometer (as red blood cell count in hematology). The clotrimazole drop (1%) was made in Behvazan Pharmaceutical Company and the lavender essential oil was prepared in Barij Essence Company. The obtained data were analyzed using descriptive statistics and the nonparametric analysis Kruskal-Wallis test and Mann-Whitney and Wilcoxon tests were used.

## 3. Findings

**Figure 1:**
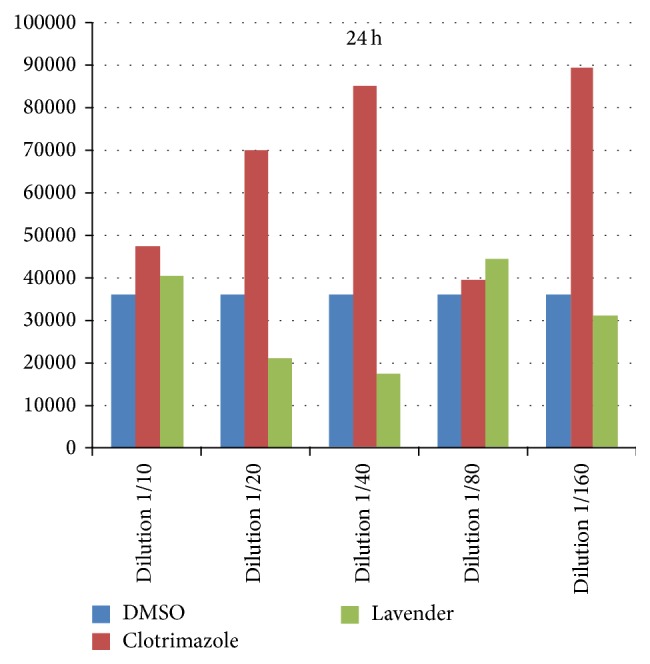
The fungus cell count in the control, clotrimazole, and lavender groups after 24 hours.

**Figure 2:**
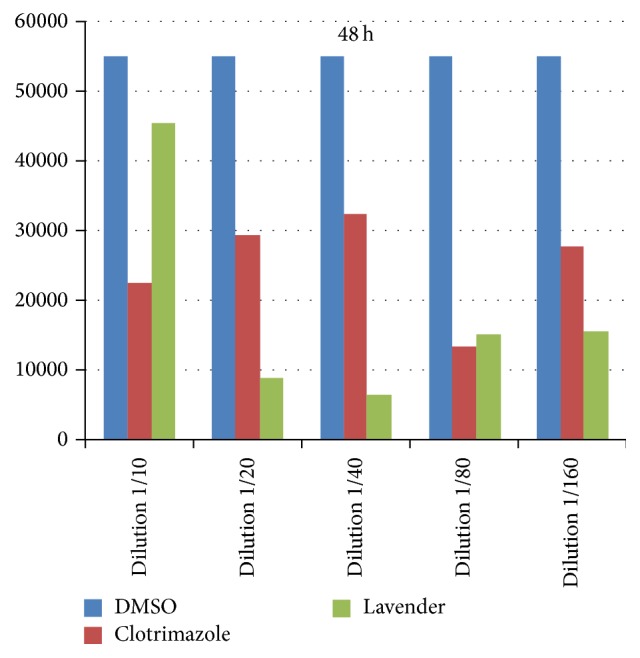
The fungus cell count in the control, clotrimazole, and lavender groups after 48 hours.

Out of the 45 samples suspected of vaginal candida infection, none of the samples had trichomoniasis in a direct test, but only 25 cases were positive for* C. albicans*. Due to the failure of the 5 cases, 20 cases of* C. albicans* obtained from patients were tested* in vitro*. Analyzing the data, it was found by normality test that the data distribution is not normal. So, the nonparametric Kruskal-Wallis test and Mann-Whitney and Wilcoxon tests were used. The comparison of the three groups of lavender, clotrimazole, and DMSO controls using the Kruskal-Wallis test in the first 24 hours of dilutions 1/40 (*P* = 0.00) and 1/160 (*P* = 0.01) indicated a significant difference between groups. With regard to the other dilutions and after 48 h, fungal cell count showed no significant differences between the three groups. The comparison between the two groups of lavender and clotrimazole using Mann-Whitney test showed that the fungal cell count after 24 hours, in dilutions of 1/20 and 1/40 and 1/160, respectively, *P* = 0.02, *P* = 0.00, and *P* = 0.01, had significant difference between the two groups. In these dilutions, during the first 24 hours, cell counts of fungi in lavender group were lower than the clotrimazole group (Figure 1), and, after 48 h, no difference was found between the groups (Figure 2). But a comparison of clotrimazole and DMSO groups showed that there is a significant difference between the two groups in dilutions of 1/40 and 1/160 and there is no significant difference between lavender and DMSO. Although, as can be seen in Figure 1, the average fungal cell count in lavender was lower than the other two groups in most dilutions, the tests showed no statistically significant differences.

The Wilcoxon test was performed separately in clotrimazole and lavender groups to compare changes between groups based on the same dilutions between 24 h and 48 h. The comparison of the clotrimazole group after 24 h and 48 h showed that there was a significant difference between the fungal cell count for dilutions of 1/10, 1/20, 1/40, 1/80, and 1/160, respectively, *P* = 0.01, *P* = 0.01, *P* = 0.00, *P* = 0.04, and *P* = 0.03, and its average in 48 h was less than 24 h. In the lavender group, a significant difference was observed between the two periods only for dilutions of 1/20 *P* = 0.02 and 1/80 *P* = 0.03. Also, in this group, the average fungal cell count was also lower after 48 h. But, for dilution 1/10, the average fungal cell count after 48 h was higher than the more dilutions. Some drugs are more effective at lower dilutions.

## 4. Discussion

With the increase in drug-resistant fungal treatment in recent years and adverse effects of antifungal agents, the need for new drugs has become important. In the present study, it has been shown that the lavender herb has antifungal activity. Studies on the antifungal effects of lavender were different. Lavender had a very weak inhibitory effect of fungus in some studies, but, in most cases, its antifungal effect was significantly positive [11–14]. Devkatte et al. [15] in a study on herbal oils as potential inhibitors of* Candida albicans* growth aimed to determine the minimum inhibitory concentration and minimum fungicidal concentration of 38 herbal oil essences. They reported that cinnamon oil was the best and had the impact of the fungicide concentration of 0.03 percent against 4 candida strains. Most oils were located in moderate group and 9 oils were the least effective in which the lavender was considered in the least effective group [15]. Mahboubi et al. [16] reported that lavender has moderate antifungal activity while other essential oils, such as thyme and geranium, have strong antifungal effects. Mahboubi showed that some essential oils were most effective against candida strains [17]. Basically, lavender oil has a long history of medicinal use in Traditional Chinese Medicine (TCM) [18] and has antimicrobial activity against fungi and bacteria [12, 19–21]. Several combinations such as linalyl acetate, linalool, butyric acid, and propionic acid are involved in this activity [11]. It was shown in a similar study that the essential oil of* Lavandula angustifolia* and its main components, linalool and linalyl acetate, can kill or inhibit the growth of fungi [18]. The data showed a significant difference between the three groups of lavender, clotrimazole, and control in higher dilutions (1/40 and 1/160) for antifungal activity. The fungal cell count in lavender group was lower than the other groups. Behmanesh et al. reported that the fungal cell count in tubes containing lavender infusion and lavender essence was lower than clotrimazole tubes. Also, the most effective antifungal effects of drugs (lower fungal cell count) were observed at higher dilutions [7]. In their study, D'Auria et al. [22] found that higher dilutions of lavender inhibit the formation of fungus and elongation of the fibers and reduce the fungal growth and its diffusion rate in the tissue.

The results of the comparison between the two groups of lavender and clotrimazole showed that fungal cell count after 24 h, for dilutions of 1/20 and 1/40 and 1/160, had significant differences. In these dilutions, the fungal cell count in lavender was lower than clotrimazole. In a similar study, it was shown that, in all the different dilutions, clotrimazole had the lowest antifungal effects and the highest fungal cell count. In this study, we found that the infusion of lavender herb and its essence as compared with clotrimazole has a higher antifungal activity against* C. albicans* [23].

Data on the comparison of clotrimazole and control groups showed significant differences in concentrations of 1/40 and 1/160 between the two groups. Gharibi et al. [24] in their study compared the concurrent use of fluconazole and clotrimazole with frequent dose in the treatment of recurrent* candidiasis *and showed that the two methods for reducing complaints and symptoms of itching, scratching, edema, erythema, and fissures were very effective two weeks after treatment (*P* = 0.00) and the symptoms of vaginal candidiasis also reduced in 6 weeks of treatment in the intervention group and had a significant difference in the control group (*P* < 0.05).

Research findings about the changes between the groups according to the same dilution but after 24 h and 48 h in the clotrimazole group showed that, for dilutions of 1/10, 1/20, 1/40, 1/80, and 1/160, there was a significant difference between the two fungal cell counts in which their average was higher in 24 h than 48 h. Also, in the lavender group, at 1/20 and 1/80 dilutions, a significant difference was observed two times and the average fungal cell count was lower after 48 h. Ghahramanlo et al. [25] conducted a similar survey and demonstrated the antifungal effects increase in acrylic resins with increasing nanoparticle concentration and time compared to contact microorganisms.

## 5. Conclusion

According to the results of this study and the information recorded in the old books of medicinal herbs of Iran, we can obtain additional information with further research to support the effectiveness of lavender.
